# METTL1 mediated tRNA m^7^G modification promotes leukaemogenesis of AML via tRNA regulated translational control

**DOI:** 10.1186/s40164-024-00477-8

**Published:** 2024-01-24

**Authors:** Pan Zhao, Lin Xia, Dan Chen, Wei Xu, Huanping Guo, Yinying Xu, Bingbing Yan, Xiao Wu, Yuxia Li, Yunfang Zhang, Xi Zhang

**Affiliations:** 1 Medical Center of Hematology, Xinqiao Hospital of Army Medical University, Chongqing, 400037 China; 2 Department of Hematology, Affiliated Hospital of North Sichuan Medical College, Nanchong, 637000 China; 3 Clinical and Translational Research Center of Shanghai First Maternity and Infant Hospital, Shanghai Key Laboratory of Signaling and Disease Research, Frontier Science Center for Stem Cell Research, School of Life Sciences and Technology, Tongji University, Shanghai, 200092 China; 4 Chongqing Key Laboratory of Hematology and Microenvironment, Chongqing, 400037 China; 5 State Key Laboratory of Trauma and Chemical Poisoning, Chongqing, 400037 China

**Keywords:** METTL1/WDR4, tRNA modification, m^7^G, AML, Translation control

## Abstract

**Background:**

RNA modifications have been proven to play fundamental roles in regulating cellular biology process. Recently, maladjusted N7-methylguanosine (m^7^G) modification and its modifiers METTL1/WDR4 have been confirmed an oncogene role in multiple cancers. However, the functions and molecular mechanisms of METTL1/WDR4 in acute myeloid leukemia (AML) remain to be determined.

**Methods:**

METTL1/WDR4 expression levels were quantified using qRT-PCR, western blot analysis on AML clinical samples, and bioinformatics analysis on publicly available AML datasets. CCK-8 assays and cell count assays were performed to determine cell proliferation. Flow cytometry assays were conducted to assess cell cycle and apoptosis rates. Multiple techniques were used for mechanism studies in vitro assays, such as northern blotting, liquid chromatography-coupled mass spectrometry (LC–MS/MS), tRNA stability analysis, transcriptome sequencing, small non-coding RNA sequencing, quantitative proteomics, and protein synthesis measurements.

**Results:**

METTL1/WDR4 are significantly elevated in AML patients and associated with poor prognosis. METTL1 knockdown resulted in reduced cell proliferation and increased apoptosis in AML cells. Mechanically, METTL1 knockdown leads to significant decrease of m^7^G modification abundance on tRNA, which further destabilizes tRNAs and facilitates the biogenesis of tsRNAs in AML cells. In addition, profiling of nascent proteins revealed that METTL1 knockdown and transfection of total tRNAs that were isolated from METTL1 knockdown AML cells decreased global translation efficiency in AML cells.

**Conclusions:**

Taken together, our study demonstrates the important role of METTL1/WDR4 in AML leukaemogenesis, which provides a promising target candidate for AML therapy.

**Supplementary Information:**

The online version contains supplementary material available at 10.1186/s40164-024-00477-8.

## Background

Acute myeloid leukemia (AML), characterized by the abnormal proliferation and differentiation of clonal myeloid precursor cells [1], is one of the most lethal hematologic malignancies and accounts for about 80% of acute leukemia in adults [2]. With the improvement of therapy regimens coupled with supportive care, the outcomes of AML patients have been largely improved in recent years [3–5]. However, the five-year survival rate of adult AML patients is still very low (lower than 40%) except for acute promyelocytic leukemia (APL) [1, 6]. Currently, there are still no encouraging effective drugs for AML therapy due to the unknown etiology, unclear pathogenesis, and high heterogeneity of AML [7]. Therefore, a better understanding of the leukaemogenesis mechanisms of AML will help to identify potential therapeutic targets and improve the clinical cure rate of AML.

Studies have shown that the initiation and progression of AML are involved in multiple-omics changes, such as genetic mutations, chromosomal translocations, and epigenetic changes including DNA methylation abnormalities, histone modification, and chromatin remodeling alterations [8, 9]. Indeed, histone acetyltransferases (HATs), histone deacetylases (HDACs), and hypomethylating agents (HMA) that target epigenetic modifications have demonstrated efficacy in the treatment of AML [4, 10]. Recently, increasing evidence revealed that the dysregulation of RNA modifications—another layer of epigenetic regulation plays essential roles in multiple human cancers, including AML [11–13]. This groundbreaking discovery potentially unveils novel and significant therapeutic possibilities for AML. Among the over 170 types of RNA modifications identified in eukaryotes [14], N6-methyladenosine (m^6^A) was the most commonly studied subtype, playing an important role in the progression of various malignant tumors [15, 16]. In AML, abnormal levels of m6A modification have been shown to affect all aspects of the disease including leukemia stem cell self-renewal [17], therapeutic resistance [18, 19], and leukemia cell proliferation [20–22] and poor survival [23]. In addition, other RNA modifications, including 2-O-methylation [24], pseudouridine (Ψ) [25], A-to-I editing [26], have emerged as effective modulators in leukaemogenesis.

N7-methylguanosine (m^7^G) modification occurred at tRNA G46, mRNA 5’ cap, and rRNAs [27]. Recent studies also report that a considerable m^7^G modification occurred at internal mRNAs [28–30]. The m^7^G modification is catalyzed by a highly conserved methyltransferase complex–methyltransferase like 1 (METTL1) and WD repeat domain 4 (WDR4) in mammalian [31, 32]. Recent studies have found that METTL1/WDR4 mediated RNA m^7^G modification plays important biological roles in human malignancies, such as cancers in the liver, colorectal, esophageal, bladder, and lung [27, 33–35]. Despite its functional importance in the aforementioned multiple solid tumors, the precise role and regulatory mechanism of METTL1/WDR4 in AML leukaemogenesis remains unclear.

In this study, we found that METTL1 and WDR4 are significantly upregulated in AML patients and the elevated expression of METTL1 and WDR4 are closely related to poor prognosis of AML patients. In addition, knockdown of METTL1 inhibits cell proliferation, promotes cell apoptosis, and arrests the cell-cycle at the G1 phase, thus suppressing AML cell progression in vitro. Mechanistically, METTL1-mediated tRNA m^7^G modification is essential for regulating intracellular global mRNA translation by affecting the biosynthesis and stability of m^7^G-harbored tRNAs. Our study illustrated the essential role of METTL1-mediated m^7^G tRNA modification in AML progression, providing new insights for the improvement of future therapeutic strategies for efficient AML treatment.

## Methods

### Specimens and ethics

A total of 53 newly diagnosed AML (as defined by the 2016 WHO Classification of myeloid neoplasms and acute leukemia [36]) patients and 6 healthy donors from the Hematology Medical Center, Xinqiao Hospital were enrolled in this study. Peripheral blood aspirates and bone marrow aspirates from the above samples were applied for subsequent RNA or protein extraction. Ethical approval for human subjects was obtained from the institutional ethics committee of Xinqiao Hospital, Army Medical University.

### Cell culture

Human AML cell lines including THP-1, MOLM-13, and HL60 purchased from American Type Culture Collection (ATCC) were cultured in RPMI 1640 medium (BI, catalog number: 011001ACS, Israel) containing 10% fetal bovine serum (BI, catalog number: 04-001-1ACS, Israel) and 1% Penicillin/Streptomycin (HyClone, catalog number: SV30010, USA). Cells were maintained at 37 °C in a cell culture incubator (Thermo Fisher Scientific, USA) with 5% CO2.

### Plasmids construction, lentiviral transfection, and siRNA transfection

For METTL1 knockdown, short hairpin RNAs targeting the METTL1 mRNA were synthesized. The forward and reverse primers were annealed to double strands DNA which was cloned into the pLKO.1 vector through EcoRI and AgeI sites. To construct METTL1 and WDR4 stable overexpression plasmids, the amplified full-length cDNAs of METTL1 and WDR4 were cloned into pWPI vector using PmeI and SpeI sites. Then, for lentivirus production, the pLKO.1 or pWPI vector was co-transfected with packaging vector psPAX2 and enveloped vector pMD2.G into 293T cells by using Lipofectamine 3000 reagent (Invitrogen, catalog number: 2175582, USA), respectively. After transfection, viruses were collected at 48 and 72 h, respectively. Then, harvested virus particles were used to infect THP-1, MOLM-13, and HL60 cells. Stable infected cells were selected using puromycin (Beyotime, catalog number: ST551, China) at 1–2 mg/ml. For transient knockdown, siRNA targeting METTL1 was transfected into THP1 cells using Lipofectamine 3000 according to the manufacturer’s protocol. Sequences of shRNA, siRNA, and overexpression PCR primers synthesized in Sangon Biotech Co., Ltd (Shang Hai, China) are listed in Additional file 5: Table S1.

### qRT-PCR and RT-PCR

Total RNAs were isolated from bone marrow mononuclear cells (BMMCs) and engineered AML cell lines with RNAiso Plus reagent (Takara, catalog number: 009109, Japan) following the manufacturer’s protocol. For qRT-PCR, the total RNA was reverse transcribed to cDNA with the M-MuLV Reverse Transcriptase kit (NEB, catalog number: M0253L, USA). qRT-PCR assay was conducted with the GoTaq qPCR Master Mix (Promega, catalog number: A6002, USA) using CFX Connect Real-Time PCR Detection System (Bio-Rad, USA). The relative mRNA expression of METTL1 and WDR4 was calculated and normalized to β-actin. The sequence of METTL1 and WDR4 primers is presented in Additional file 5: Table S1. To evaluate expression levels of tsRNA, RT-PCR was conducted. In brief, the total RNA was polyadenylated and converted to cDNA in the M-MuLV Reverse Transcriptase Reaction System with a unique adaptor, then the cDNA was amplified using the universal adaptor and specific tsRNA primers. The PCR product was checked with a 3% agarose gel. The sequence of tsRNA primers is presented in Additional file 5: Table S1.

### Cell proliferation assay

CCK-8 assay and cell count assay were used to determine cell proliferation. For CCK8 assay, 3 × 10^3^ cells were seeded into the 96-well plates and incubated for several days in a row. 10 μl CCK-8 solution (Beyotime, catalog number: C0043, China) was added to each well at the same time pointed each day, and cells were incubated for 2 h. Then, the optical density (OD) values in the wavelength of 450 nm were determined to assess cell viability using the microplate reader Varioskan FLASH (Thermo Fisher Scientific, USA). For cell count assay, 1.2 × 10^4^ cells were seeded into the 24-well plates and counted on day 2 and day 4 after seeding using cellometer auto 2000 cell viability counter (Nexcelom, USA).

### Cell cycle assay

Cell cycle assay was performed using PI/RNase Staining buffer (BD, catalog number: 550825, USA) in accordance with the manufacturer’s instruction. Briefly, 2 × 10^6^ cells were harvested, washed with PBS, and fixed by cold 70% ethanol overnight at 4 °C. Next, the fixed cells were washed again and stained with a PI/RNase solution for 30 min. Finally, the fixed and stained cells were analyzed by flow cytometry using CytoFLEX (Beckman Coulter, USA).

### Cell apoptosis assay

1 × 10^6^ cells per well were seeded into a 6-well plate and cultured for 48 h. Then, apoptotic cells were detected by CytoFLEX (Beckman Coulter, USA) with the Annexin V-FITC Apoptosis Detection Kit (Beyotime, catalog number: C1062L, China) according to manufacturer’s instructions. The FlowJo software version 7.6 (FlowJo LLC, Ashland, KY, USA) was employed for data analysis.

### Northern blot

Northern blotting was performed as previously described [37]. Briefly, 3 μg total RNAs extracted from engineered AML cell lines were mixed with 2 × RNA Loading Dye (NEB, catalog number: B0363S, USA), denatured at 95 °C for 5 min, and electrophoresed on 15% urea-PAGE gels in 1 × TBE buffer (Invitrogen, catalog number: B540024-0001, USA). Then, the gels were stained with SYBR® GOLD Nucleic Acid Gel Stain (Invitrogen, catalog number: S11494, USA), imaged, and transferred onto nylon membranes (Roche, catalog number: 11417240001, Switzerland). Next, the membranes were cross-linked with Ultraviolet light and incubated with digoxigenin (DIG) labeled oligonucleotide probes (Sangon, China) overnight. The next day, the membranes were incubated with DIG antibody (Anti-Digoxigenin—AP Fab fragments, Roche, catalog number: 11093274910, Switzerland) and photographed using the Bio-Rad system (USA). DIG-labeled probes used for the northern blot are presented in Additional file 5: Table S1.

### Western blot

Protein was extracted from BMMCs and engineered AML cell lines with NP-40 Lysis Buffer (Beyotime, catalog number: P0013F, China), supplemented with 1 mM PMSF (Beyotime, catalog number: ST506, China). Protein concentrations were assessed by the Enhanced BCA Protein Assay Kit (Beyotime, catalog number: P0010, China). For Western blot, 40 μg protein was separated on SDS-PAGE gels and transferred onto nitrocellulose membranes (Millipore, catalog number: HATF00010, USA). After blocking with 5% skimmed milk, the membranes were incubated with primary antibody against METTL1 (Proteintech, catalog number: 14994-1-AP, China), WDR4 (Santa Cruz, catalog number: sc-100894, USA), BCL2 (Proteintech, catalog number: 60178-1-1, China), Caspase3 (Proteintech, catalog number: 19677-1, China) and β-actin (Proteintech, catalog number: 66009-1-Ig, China) at 4 °C overnight. Whereafter, the membranes were incubated with Horseradish peroxidase (HRP)-conjugated goat anti-rabbit immunoglobulin G (Proteintech, catalog number: SA00001:15, China) and HRP-conjugated goat anti-mouse immunoglobulin G (Beyotime, catalog number: A0216, China) secondary antibody. Finally, Blots were scanned using Bio-Rad system (USA).

### Isolation of tRNA, tsRNA, and mRNA

Total RNA was extracted from METTL1 knockdown (shMETTL1) and METTL1 control (shNC) THP1 cells using RNAiso Plus reagent and then separated on 15% Urea-PAGE gels. After staining with SYBR® Gold Nucleic Acid Gel Stain (Invitrogen, catalog number: S11494, USA), ~ 80 nt tRNAs and 30–40 sized tsRNAs were excised and extracted from the gels according to Low Range ssRNA Ladder (NEB, catalog number: N0364S, USA). mRNA was extracted from total RNA using NEB Next®Poly(A) mRNA Magnetic Isolation Module (NEB, catalog number: E7490L, USA) according to the manufacturer’s protocol.

### Decapping of mRNA

Decapping of mRNA was performed with Tobacco Decapping Plus 2 (#94, Enzymax) as described previously [29]. Briefly, 3 μg poly(A) + RNAs were incubated in 50 μl reaction system including 5 μl 10 × Decapping Reaction Buffer (100 mM Tris–HCl pH 7.5, 1.0 M NaCl, 20 mM MgCl2, 10 mM DTT), 1uL 50 mM MnCl2, 1 μl RNase Inhibitor (Takara, catalog number: 2311A, Japan), 4 μl Tobacco Decapping Plus 2 enzyme, and nuclease-free water. The reaction was incubated at 37 °C for 2 h. Decapped RNAs in the solution were then extracted with RNA Clean concentrator-25 (Zymo Research, R1018, USA) according to the manufacturer’s protocol.

### Liquid chromatography-coupled mass spectrometry for the quantitative analysis of tRNA and mRNA modifications

Liquid chromatography-coupled mass spectrometry (LC–MS/MS) was conducted as previously described [38]. Briefly, the RNAs concentration was measured, and 100 ng RNAs were digested in a 30 μl enzymolysis system including 3 μl 10 × buffer (250 mM Tirs-HCl, pH 8.0; 5 mM MgCl2 and 0.5 mg/ml BSA), 1 IU Benzonase (Sigma-Aldrich, catalog number: E8263, USA), 0.2 IU alkaline phosphatase (Sigma-Aldrich, catalog number: P5521, USA) and 0.05 IU phosphodiesterase I (USBiological, catalog number: P4072, USA) at 37 °C for 3 h. The enzymolysis samples then were centrifuged to obtain the mononucleotides using Nanosep® 3K spin filter (Pall Corporation, catalog number: 0D003C34, USA). Next, the mononucleotides were used to determine RNA modification levels by Acquity-UPLC I-class carrying Xevo-TQ-S mass spectrometry system (Waters Corporation, USA) with multiple reaction monitoring (MRM) scan model. LC–MS raw data were acquired and processed by MassLynx (version 4.1) software. The percentage of each modified ribonucleoside was normalized to the total amount of quantified ribonucleosides with the same nucleobase.

### tRNA stability analysis

To analyze the stability of tRNA, 15 ng tRNAs extracted from knockdown (shMETTL1) and METTL1 control (shNC) THP1 cells were incubated in 9ul RPMI 1640 with 0.0003 μl RNaseA/T1 (2 mg/ml of RNase A and 5000 U/ml of RNase T1) or 9 μl RPMI 1640 alone at 37 °C for 5 min. The samples were then refrigerated on ice and mixed with 2 × RNA Loading Dye (NEB, catalog number: B0363S, USA). Next, 4 μl mixtures were run in 10% native PAGE gels at 4 °C. Lastly, the gels were imaged using the Bio-Rad system (USA) after stained with SYBR Gold.

### Small RNA library construction, sequencing, and analysis

Small RNA library construction and sequencing were performed by the Beijing Genomics Institute (BGI, Beijing, China). Briefly, small RNAs of size 15–50 nt were excised and recovered from the total RNAs of knockdown (shMETTL1) and METTL1 control (shNC) THP1 cells and METTL1-OE/METTL1-CON HL-60 cells, then ligated to 3' and 5' adapters. Subsequently, the adapter ligated small RNAs were reverse transcribed and PCR amplified to obtain enriched cDNA fragments. Finally, cDNA fragments were sequenced using Illumina-HiSeq 2500 platform. Raw reads were processed using SPORTS (v1.1) and the param was set as follows: (1) sequencing reads shorter than 15 nt or longer than 45 nt were discarded, (2) reads allowed to have a mismatch when mapped to sncRNA references. sncRNA references used in this paper include genome, miRNA reference, rRNA and YRNA reference, genomic tRNA reference, mitochondrial tRNA reference, piRNA reference, the non-coding RNA reference. Additionally, a tsRNA was considered to be significantly differentially expressed when the p value is <  = 0.05 and the absolute value of log_2_ fold change >  = 1.

### Public database analysis

Briefly, the microarray data were downloaded from NCBI Gene Expression Omnibus (GEO) under the accession GSE58831, GSE6891, GSE9476, and GSE15061. Then, METTL1 and WDR4 gene expression levels were estimated among FAB types of acute myeloid leukemia with healthy control. The Cancer Genome Atlas (TCGA) LAML datasets and DiseaseMeth datasets were used for survival, DNA methylation, and gene expression correlation analysis between METTL1 and WDR4.

### Transcriptome sequencing and data analysis

After extraction of total RNA from engineered AML cells (THP-1), transcriptome sequencing was performed by Personalbio Technology Co., Ltd (Shanghai, China) according to the manufacturer’s protocol. Firstly, mRNAs were enriched with polyA tails by Oligo (DT) magnetic beads from total RNA. Then mRNAs were randomly interrupted, linked with connectors, and reverse transcribed to cDNA for library construction. Finally, pair-end sequencing was conducted based on high-throughput sequencing platform (illumina Novaseq 6000). For the data processed, (1) the raw sequencing data were filtered by Trimmomatic (v0.38) to remove the 3′ and 5′ adapter and low-quality reads [39], (2) high-quality sequences were mapped to the human genome (hg38, human genome assembly GRCh38) using Hisat2 (v2.1.0) [40], (3) gene expression level (TPM) was quantified by StringTie (v2.2.0) [41].

### Quantitative proteomics and data analysis

Quantitative proteomics was performed by Novogene Co., Ltd (Bei jing, China) according to the manufacturer’s protocol. Firstly, total proteins were extracted from engineered AML cells (THP-1) and digested with trypsin. Then the protein samples labelled with TMT reagent, combined, fractionated, and split for quantitative analysis using UHPLC-MS/MS on an EASY-nLCTM 1200 UHPLC system (Thermo Fisher, Germany) coupled with an Q ExactiveTM HF-X (Thermo Fisher, Germany). Raw files are directly imported into Proteome Discoverer 2.5 software for database retrieval, peptide spectrum and protein quantification. The proteins whose quantitation significantly different between experimental and control groups, (*p* < 0.05 and FC < 0.83), were defined as differentially expressed proteins (DEP). DEPs were used for Gene Ontology (GO) enrichment analysis using the interproscan program against the non-redundant protein database (including Pfam, PRINTS, ProDom, SMART, ProSite, PANTHER).

### tRNA and tsRNA transfection

One mg tRNAs, 400 ng tsRNAs isolated from knockdown (shMETTL1) and METTL1 control (shNC) THP1 cells were transfected into 1 × 10^6^ THP1 cells using Lipofectamine™ 3000 Reagent (Invitrogen, catalog number: L300075, USA) according to the instructions. After 48 h of transfection, the cells were harvested for subsequent OPP labeling.

### Protein synthesis measurement using OPP

The OPP labeling assay was performed using Click-iT® Plus OPP Protein Synthesis Assay Kits (Life technology, catalog number: C10456, USA) according to manufacturer’s instructions. In brief, 2 × 10^6^ cells were harvested, resuspended in 1 ml medium containing 20 μM Click-iT® OPP, and incubated at 37 °C for 30 min. After incubation, cells were fixed with 3.7% formaldehyde and permeabilized with 0.5% Triton®X-100 for 15 min at room temperature, respectively. Next, the cells were incubated with 1 ml Click-iT® Plus OPP reaction cocktail consisting of 880 μl Click-iT® OPP reaction buffer, 20 μl Copper Protectant, 2.5 μl Alexa Fluor® picolyl azide and 100 μl Click-iT® Reaction Buffer for 30 min at room temperature. Finally, flow analysis was conducted using a BD LSR Fortessa Flow Cytometer (BD, USA) with the Texas Red® channel for Alexa Fluor® 594. The FlowJo software version 7.6 (FlowJo LLC, Ashland, KY, USA) was used for data analysis.

## Results

### METTL1 and WDR4 are upregulated in AML and associated with poor prognosis

**Fig. 1:**
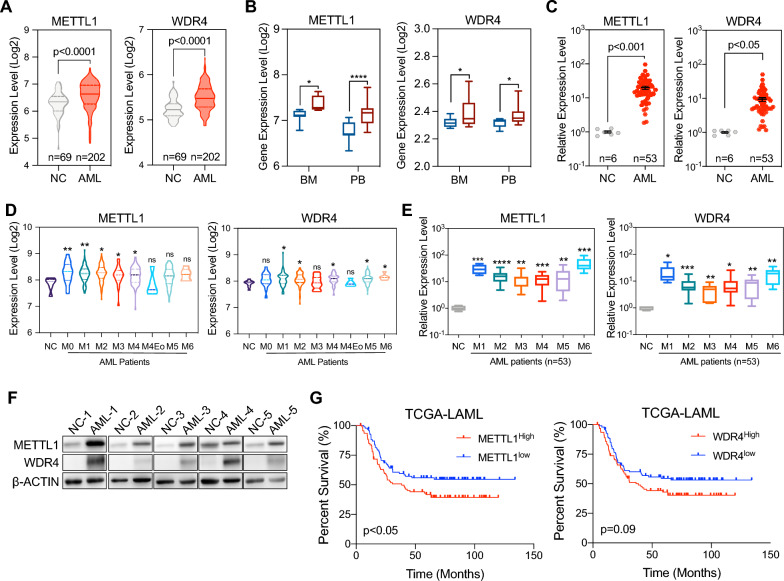
METTL1/WDR4 were upregulated and associated with poor prognosis of AML patients. **A** Expression Level of METTL1 and WDR4 in healthy individuals (NC) and AML patients from the GEO dataset. **B** Comparison of METTL1 and WDR4 expression in bone marrow (BM) and peripheral blood (PB) samples between AML patients (red box) and healthy individuals (blue box) from the GEO dataset. **C** Expression level of METTL1 and WDR4 in bone marrow mononuclear cells between healthy donors (NC) and AML patients from our center. **D** Comparison of METTL1 and WDR4 expression in healthy individuals and various subtypes of AML patients in the GEO dataset. **E** Expression level of METTL1 and WDR4 between healthy donors and various subtypes of AML patients from our center. **F** Representative bands of METTL1 and WDR4 examined by western blot assay in healthy donors and AML patients from our center. **G** Correlation between METTL1/WDR4 expression and overall survival of AML patients according to data from TCGA. Data were presented as mean ± SEM (Student’s t test, *p < 0.05, **p < 0.01, ***p < 0.001, ****p < 0.0001, ns: not significant)

To assess the expression of METTL1/WDR4 in AML, publicly available datasets involving healthy individuals and newly diagnosed AML patients were collected from the NCBI GEO database. Transcriptome analysis of bone marrow cells showed that the expression levels of METTL1 and WDR4 were significantly increased in AML patients compared to healthy individuals (Fig. 1A). Further comparative analyses of public datasets and qRT-PCR validation of newly diagnosed AML patients and healthy donors from Xinqiao Hospital revealed that METTL1 and WDR4 were significantly upregulated in both bone marrow and peripheral blood samples (Fig. 1B, C). For subtype analysis, METTL1 and WDR4 exhibited upregulation in nearly all FAB subtypes, especially in M1-, M2-, M4-, M5- and M6-subtypes (Fig. 1D, E) of AML. Additionally, we reanalyzed the abundance of METTL1/WDR4 complex in AML patients with the translocation and mutation subtypes according to the 5th WHO classification standard. In samples with complete clinical data, we observed an upregulation expression of METTL1 and WDR4 across most WHO subtypes compared to healthy donors (Additional file 1: Fig. S1A, -B). We also analyzed the public datasets to confirm our observation in patients and the results showed similar conclusion to our experimental data (Additional file 1: Fig. S1C, D). Western blot assays also confirmed that METTL1 and WDR4 were expressed higher in the bone marrow mononuclear cells of AML patients (Fig. 1F). Moreover, using the acute myeloid leukemia (LAML) datasets from the Cancer Genome Atlas (TCGA), we found that poor patient survival was shown in AML patients which had significantly high expression levels of METTL1 or WDR4 (Fig. 1G), indicating potential functions of METTL1 and WDR4 in the regulation of AML progression. Overall, these data reveal that METTL1 and WDR4 are significantly up-regulated in AML patients and associated with AML progression, suggesting the potential clinical significance of METTL1 and WDR4 in AML.

### METTL1 knockdown inhibits human AML cell survival and growth

**Fig. 2:**
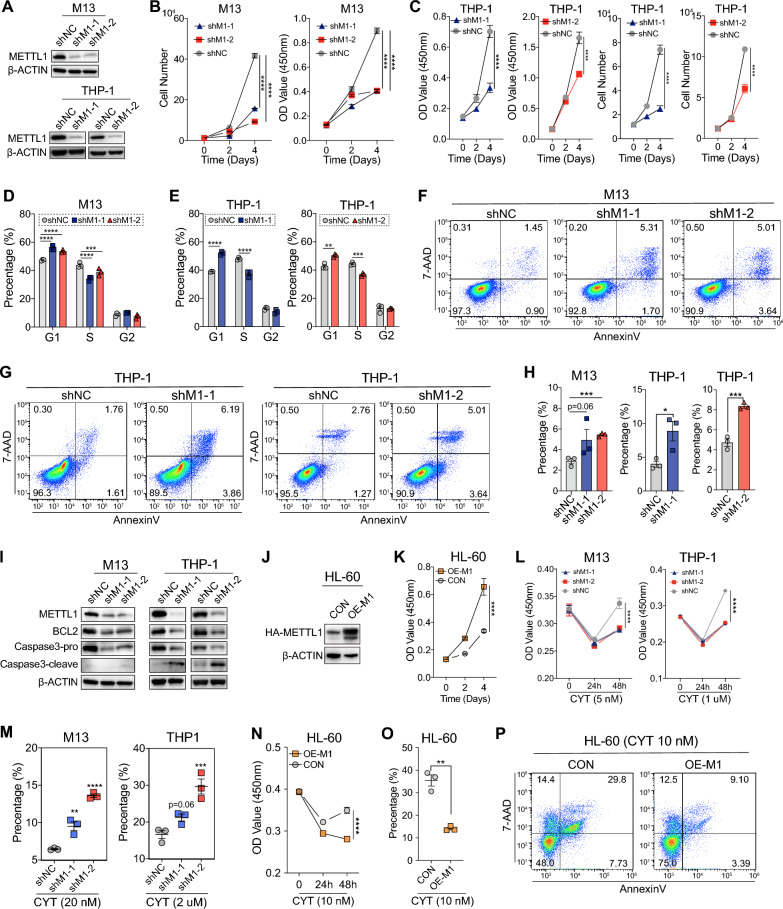
METTL1 knockdown inhibits the survival and growth of human AML cells. **A** Validation of the knockdown effect of METTL1 by western blot in MOLM-13 (M13) and THP-1 cells. **B**, **C** Knockdown of METTL1 suppressed cell proliferation of THP-1 and M13 cells. **D**, **E** Knockdown of METTL1 induced an increased ratio of G1-phase cells and decreased the ratio of S-phase cells. **F**–**H** Knockdown of METTL1 induced an increased cell apoptosis in THP-1 and M13 cells. **F**, **G** Representative images of flow cytometry analysis. **H** Quantification data. **I** Knockdown of METTL1 induced changes of the apoptosis-related proteins in THP-1 and M13 cells. **J** Validation of the overexpression effect of METTL1 by western blot in HL60 cells. **K** Overexpression of METTL1 promoted cell proliferation of HL60 cells. **L** Knockdown of METTL1 suppressed proliferation of THP-1 and M13 cells while treated with cytarabine (CYT). **M** Knockdown of METTL1 increased cell apoptosis in THP-1 and M13 cells while treated with CYT for 48 h. **N** Overexpression of METTL1 increased cell growth rates of HL60 cells while treated with CYT. **O**, **P** Overexpression of METTL1 decreased cell apoptosis in HL60 cells while treated with CYT for 48 h. **O** Quantification data. **P** Representative images of flow cytometry analysis. shNC: METTL1 control, shM1: METTL1 knockdown, CON: METTL1 control, OE-M1: METTL1 overexpression. Data were presented as mean ± SEM (Student’s t test, *p < 0.05, **p < 0.01, ***p < 0.001, ****p < 0.0001)

To investigate the role of METTL1 in AML, we used short hairpin RNA systems to knock down the expression level of METTL1, which included a negative control (shNC) and two independent shMETTL1 (shMETTL1-1: shM1-1 and shMETTL1-2: shM1-2), which showed efficient knockdown of the protein levels of METTL1 in THP-1 and MOLM-13 AML cell lines (Fig. 2A). As expected, knockdown of METTL1 inhibited cell growth rate in above AML cells (Fig. 2B, C). Cell cycle analyses showed a significant increase of G1-phase cells and reduction of S-phase cells after METTL1 knockdown in AML cells (Fig. 2D, E), indicating that knockdown of METTL1 induced cell cycle arrest in the G1 phase. Moreover, Annexin V/7-AAD staining showed that knockdown of METTL1 induced cell apoptosis in AML cells (Fig. 2F–H). The western blot showed that METTL1 knockdown led to the downregulation of BCL2 and promoted the cleavage of Caspase3, which increased the apoptosis rate in AML cells (Fig. 2I). Conversely, overexpression of METTL1 in HL60 AML cells promoted cellular proliferation (Fig. 2J, K). When subjected to cytarabine (CYT) treatment, METTL1 knockdown AML cells exhibited a significantly diminished proliferation capacity and increased susceptibility to cytarabine-induced cell death (Fig. 2L, M and Additional file 2: Fig S2A, B). Conversely, METTL1 overexpressing HL60 cells treated with cytarabine exhibited significantly accelerated growth rates and decreased cell apoptosis (Fig. 2N–P). Overall, these results indicate that METTL1 is essential for the survival of AML cells.

### The expression of METTL1 in AML was regulated by DNA methylation

**Fig. 3:**
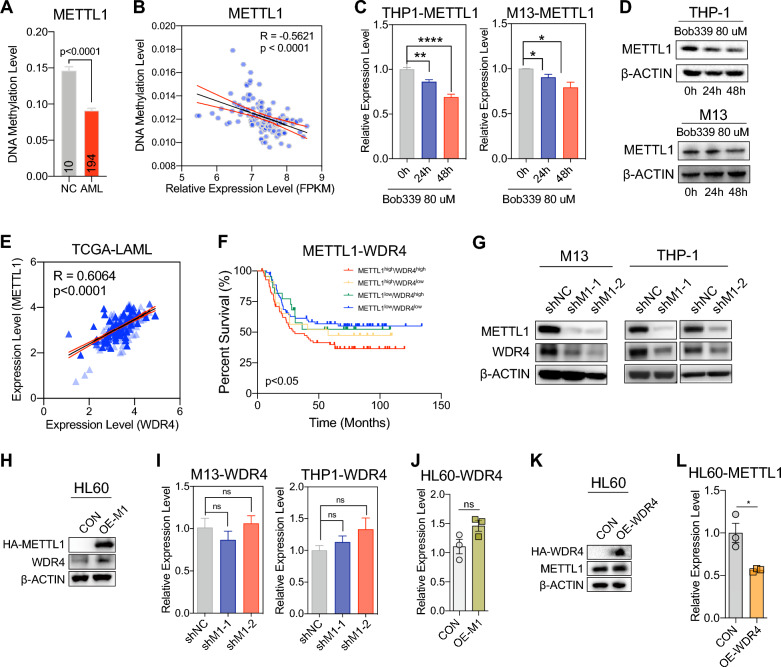
DNA methylation regulates METTL1 expression and the correlation of METTL1 and WDR4 in AML cells. **A** Comparison of METTL1 DNA methylation level in healthy individuals (NC) and AML patients from TCGA dataset. **B** Correlation between mRNA expression level (FPKM) and DNA methylation level of METTL1 in AML patients from TCGA dataset. **C**, **D** The mRNA (**C**) and protein (**D**) expression level of METTL1 in AML cells while treated with bobcat339 hydrochloride. **E** Correlation between METTL1 and WDR4 mRNA levels in AML patients from TCGA dataset. **F** Correlation between the METTL1 and WDR4 expression levels and overall survival of AML patients from TCGA dataset. **G** The protein expression level of WDR4 in METTL1 knockdown (shM1) and METTL1 control (shNC) AML cells. **H** The protein expression level of WDR4 in METTL1 over-expression (OE-M1) and METTL1 control (CON) HL60 cells. **I** The mRNA expression level of WDR4 in METTL1 knockdown (shM1) and METTL1 control (shNC) AML cells. **J** The mRNA expression level of WDR4 in METTL1 over-expression (OE-M1) and METTL1 control (CON) HL60 cells. **K**, **L** The protein (**K**) and mRNA (**L**) expression level of METTL1 in WDR4 over-expression (OE-WDR4) and WDR4 control (CON) HL60 cells. Data were presented as mean ± SEM (Student’s t test, *p < 0.05, **p < 0.01, ****p < 0.0001)

Previous data suggested that the expression of METTL1 might be regulated by DNA methylation [42]. To investigate why METTL1 maintained a high expression status in AML cells, we collected DNA methylation array data (450k) of AML from the database DiseaseMeth and TCGA. Interestingly, we found that the DNA methylation level of METTL1 (chr12:58165414–58167914) in AML patients was lower than in healthy control (DiseaseMeth data, Fig. 3A). Based on the gene information from the National Center for Biotechnology Information (NCBI), METTL1 is located on exon 1 of chromosome 12, specifically at position 58162254–58165888 (−), and this DNA methylation region is approximately situated within 2 kilobases (kb) from the transcription start site (TSS), thereby it can be classified as in the gene promoter region. Moreover, the DNA methylation level of METTL1 was negatively correlated with METTL1 mRNA expression (TCGA data, Fig. 3B). By applying the cytosine-based TET enzyme inhibitor (bobcat339 hydrochloride), which targets the overall DNA demethylation levels, to treat AML cells, we found that the transcription level of METTL1 was significantly decreased in AML cell lines after 24 h and 48 h treatment of bobcat339 hydrochloride, together with the downregulation of METTL1 proteins (Fig. 3C, D), suggesting that the DNA demethylation in METTL1 gene’s promoter region that regulated by *Tet* family might play a major role in regulating METTL1 activation in AML cells.

### Collaborative expression of METTL1 and WDR4 in AML cells is guided by METTL1

Previous studies have shown that METTL1 and WDR4 work together to add m^7^G modification in RNAs, and showed collaborative expression patterns in various cancers, including AML [35]. To further investigate the regulating mechanism of the collaborative expression of METTL1 and WDR4 in AML, we first analyzed the transcriptome datasets of acute myeloid leukemia (LAML) from the Cancer Genome Atlas (TCGA) and confirmed that the transcription levels of METTL1 were positively correlated to WDR4 (r = 0.6064, p < 0.0001, Fig. 3E). Moreover, AML patients expressed both high METTL1 and WDR4 indicate worst prognosis (METTL1^high^WDR4^high^, Fig. 3F), suggesting that a positive collaborative expression pattern existed between METTL1 and WDR4. Further analysis showed that the protein level of WDR4 significantly decreased upon METTL1 knockdown, and increased along with METTL1 overexpression (Fig. 3G, H). However, the mRNA expression level of WDR4 showed unobvious altered upon METTL1 knockdown or overexpression in AML cells (Fig. 3I, J), indicating that the regulation of WDR4 via METTL1 may occur at the post-transcriptional level. Noteworthy, alteration of the expression of WDR4 has a slight effect on the expression level of METTL1 neither at mRNA nor at the protein level in AML cells (Fig. 3K, L). These results suggested that the collaborative expression of METTL1 and WDR4 in AML cells is guided by METTL1 via a post-transcriptional regulation mechanism.

### Knockdown of METTL1 leads to the reduction of cellular m^7^G modification abundance on tRNA and mRNAs.

**Fig. 4:**
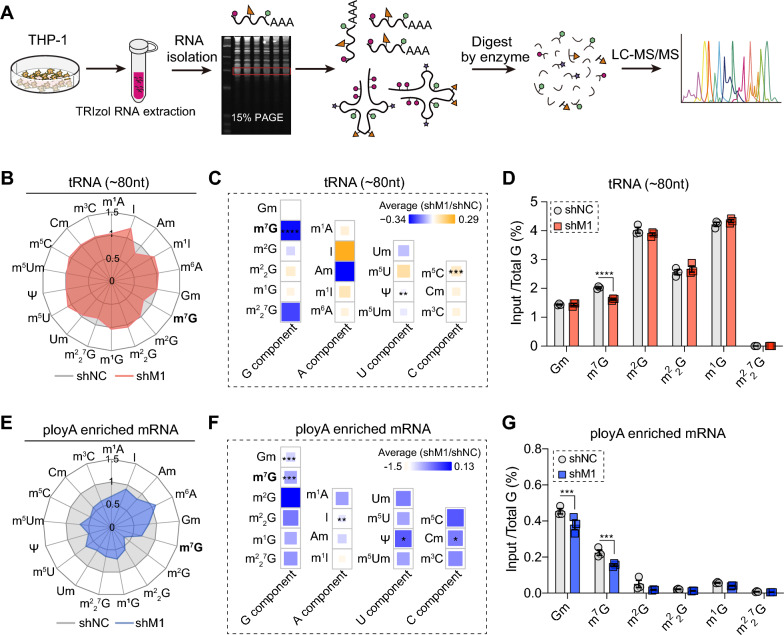
Knockdown of METTL1 leads to a reduction in the abundance of cellular m^7^G modification on tRNA and mRNA in THP-1 cells. **A** Experimental procedures for detecting RNA modifications in THP-1 cells. **B**, **C** Radar map (**B**) and heatmap (**C**) showed the comparison of RNA modification levels in tRNA-enriched fragments (tRNA, ~ 80nt) in METTL1 knockdown and control THP-1 cells. **D** Comparison of m^7^G modification level on tRNA in METTL1 knockdown and control THP-1 cells. **E**, **F** Radar map (**E**) and heatmap (**F**) showed the comparison of RNA modification levels in mRNA in METTL1 knockdown and control THP-1 cells. **G** Comparison of m^7^G modification level on mRNA in METTL1 knockdown and control THP-1 cells. Data were presented as mean ± SEM (Student’s t test, ***p < 0.001, ****p < 0.0001)

To confirm whether METTL1 could determine the abundance of m^7^G modification on both tRNA and mRNA in AML cells, we applied LC–MS/MS-based high-throughput RNA modification quantitative detection platform to detect m^7^G RNA modification status in METTL1 knockdown AML cells. Firstly, we isolated the tRNA-enriched fragments (~ 80 nt, mainly tRNAs, tRNA) from AML cells with METTL1 or without METTL1. By performing LC–MS/MS platform, we quantified 18 types of RNA modifications on tRNA and mRNA (Fig. 4A). Compared to the controls, the level of m^7^G on tRNA was significantly decreased in METTL1 knockdown AML cells, while the abundances of other types of RNA modification were almost not affected (Fig. 4B–D and Additional file 3: Fig. S3A), indicating that METTL1 specifically affects m^7^G abundance on tRNA in AML cells. Recently studies showed that mRNA also contained a considerable abundant internal m^7^G modification in mammalian cells [28, 29], so we next to detect the m^7^G level on poly(A) enriched mRNAs. Since the mRNA only takes a small proportion of total RNAs (~ 3–7%) [43], and the mRNAs usually contain three m^7^G caps on the 5’end of each mRNA molecular [44], we performed two-round poly (A) enrichment experiment to remove the contamination from other RNAs and performed twice de-capping experiments to largely remove the m^7^G cap on the 5’ end of mRNAs to focus on the abundance alteration of internal m^7^G on mRNAs. The results showed that the level of m^7^G on poly(A) enriched mRNA was significantly decreased in METTL1 knockdown AML cells, while the levels of m^2^G, m^1^G, m^2^_2_G, m^2^_2_^7^G were not affected (Fig. 4E–G and Additional file 4: Fig. S4B). Interestingly, different to RNA modification abundance on tRNAs, the level of Gm was also decreased in poly(A) enriched mRNAs with METTL1 knockdown (Fig. 4F, G), suggesting that there might be co-regulation mechanism between Gm and m^7^G on mRNA in AML cells which need further investigation.

### METTL1 knockdown reduced cellular nascent protein synthesis in AML cells via dysregulation of tRNA epitranscriptome

**Fig. 5:**
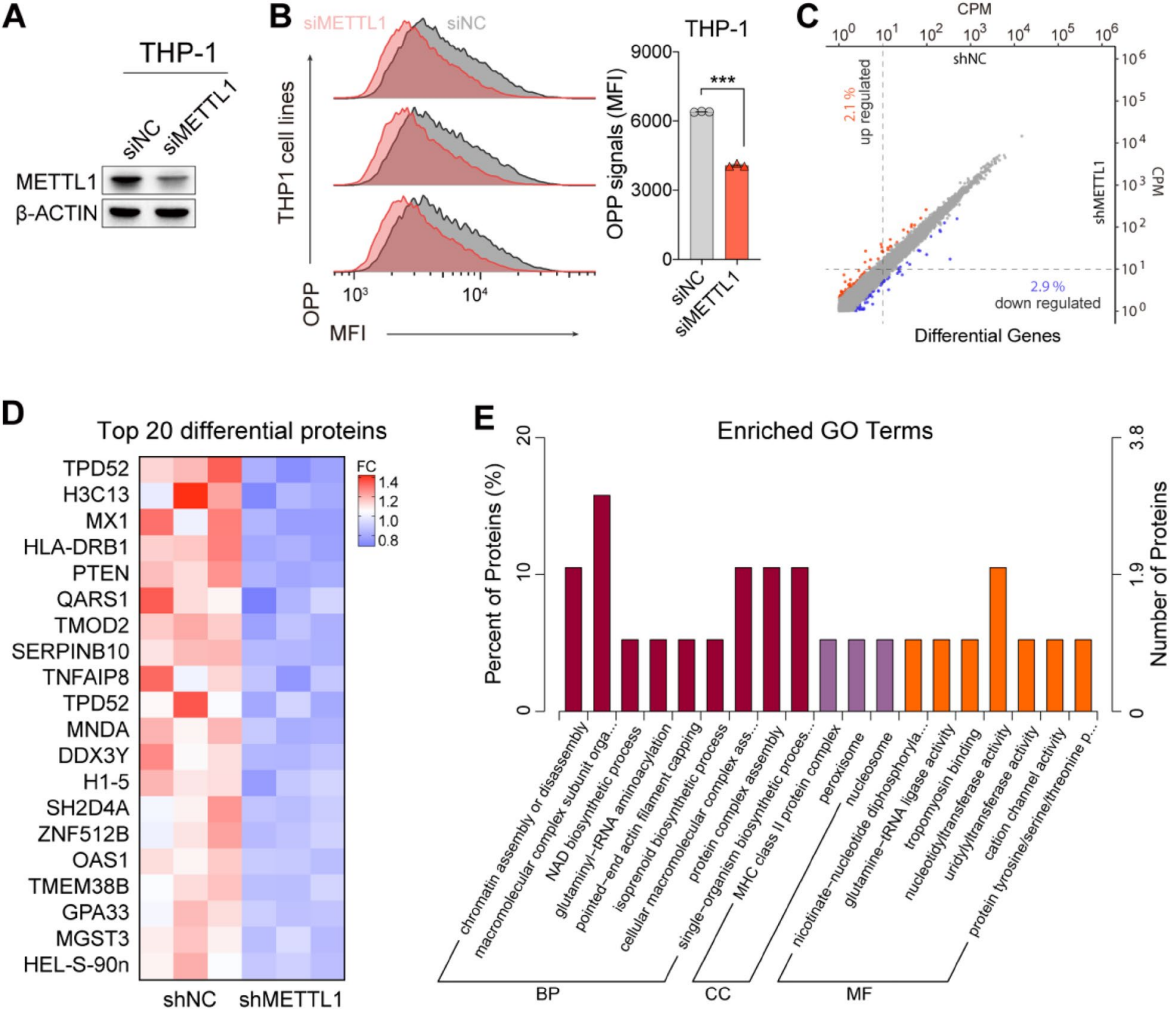
METTL1 knockdown inhibits cellular nascent protein synthesis in THP-1 cells. **A** Western blotting confirmation of METTL1 knockdown using siRNAs in THP-1 cells. **B** The level of OPP means fluorescence intensity (MFI) in METTL1 knockdown (siMETTL1) and METTL1 control (siNC) THP-1 cells. Left panels: representative images. Right panels: quantification data. **C** Transcriptome sequencing analysis of gene changes in THP-1 cells upon METTL1 knockdown. **D** The top 20 down-regulation proteins in METTL1 knockdown THP1 cells compared to the controls. **E** GO enrichment analysis of down-regulation proteins in METTL1 knockdown THP-1 cells. Data were presented as mean ± SEM (Student’s t test, ***p < 0.001)

Recently studies showed that the deletion of METTL1 could decrease the translation efficiency of m^7^G modified transcripts in mammalian cells and highlight mRNA internal m^7^G as a novel epitranscriptome marker with regulatory roles in translation [28, 29]. Moreover, studies also showed that the METTL1/WDR4-mediated m^7^G on tRNA methylome is also required for normal mRNA translation in mESC and regulates ESC self-renewal and differentiation [45]. Since our data showed that the knockdown of METTL1 could decrease the levels of m^7^G both on poly(A) enriched mRNAs and total tRNA in AML cells, we next to investigate whether the METTL1 knockdown induced reduction of m^7^G abundance in AML cells could affect cellular translation efficiency. We performed an O-propargyl-puromycin (OPP) labeling experiment to detect the overall nascent protein biogenesis in AML cells. The results showed that knockdown of METTL1 by siRNA indeed reduced the level of OPP mean fluorescence intensity (MFI) in AML cells (Fig. 5A, B), indicating that the nascent protein synthesis and global mRNA translation were inhibited in METTL1 knockdown AML cells. However, the transcriptome sequencing showed few gene alterations in METTL1 knockdown AML cells (Fig. 5C), the result indicating that the observed inhibition of nascent protein synthesis in METTL1 knockdown AML cells may not be predominantly influenced by alterations at the transcriptome level. Moreover, quantitative proteomics experiment revealed that the downregulated proteins induced by knockdown of METTL1 were enriched in the protein translation process (e.g., protein complex assembly, protein complex, protein dephosphorylation) and a series of enzyme activity (protein tyrosine/serine/threonine phosphatase activity, glutamine-tRNA ligase activity, glutaminyl-tRNA aminoacylation) (Fig. 5D, E), which consist with the above results that METTL1 regulate the global translation in AML cells.

**Fig. 6:**
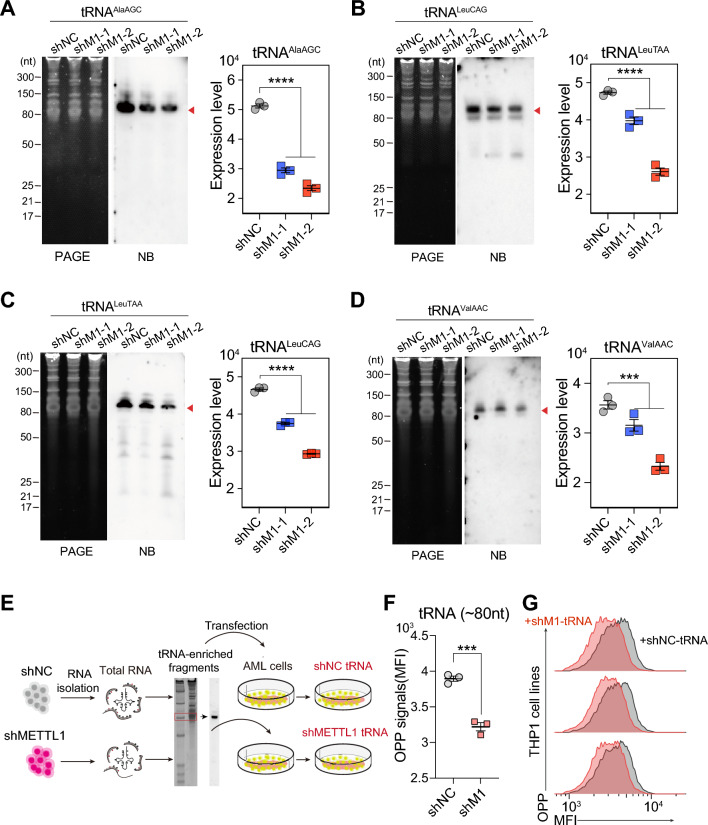
METTL1 knockdown leads to tRNA dysregulation for nascent protein synthesis inhibition. **A**–**D** Representative tRNAs level examined by northern blot in METTL1 knockdown (shM1) and METTL1 control (shNC) THP-1 cells. Left: NB images. Right: quantification data. **E** Experimental procedures for OPP labeling experiments in **F**, **G**. **F**, **G** The level of OPP means fluorescence intensity (MFI) in THP-1 cells transfected with tRNAs extracted from METTL1 knockdown (shM1) or METTL1 control (shNC) THP-1 cells, respectively. **F** Quantification data. **G** Representative images of flow cytometry analysis. Data were presented as mean ± SEM (Student’s t test, ***p < 0.001, ****p < 0.0001)

To further confirm whether the translation inhibition in METTL1 knockdown AML cells was induced by m^7^G dysregulated tRNA methylome, we first performed northern blot to detect the levels of some selected tRNAs in AML cells, the data showed that the levels of tRNA^Ala^, tRNA^Val^, and tRNA^Leu^ were significantly decreased in METTL1 knockdown AML cells compared to METTL1 control AML cells (Fig. 6A–D), suggesting that the absence of METTL1 mediated tRNA m^7^G might affect the cellular tRNA spectrum in AML cells. Further OPP labeling experiments showed decreased levels of OPP mean fluorescence intensity (MFI) in AML cells transfected with tRNAs isolated from METTL1 knockdown cells compared to those from METTL1 control cells (Fig. 6E–G), suggesting that the m^7^G dysregulated tRNA methylome also affected the translation function of cellular tRNA pools. Taken together, the above results suggest that METTL1-mediated tRNA m^7^G modification could regulate cellular tRNA pools and tRNA epitranscriptome, which further leads to the dysregulation of global mRNA translation in AML cells.

### METTL1 promotes the biogenesis of tRNA derived small RNA in AML cells

**Fig. 7:**
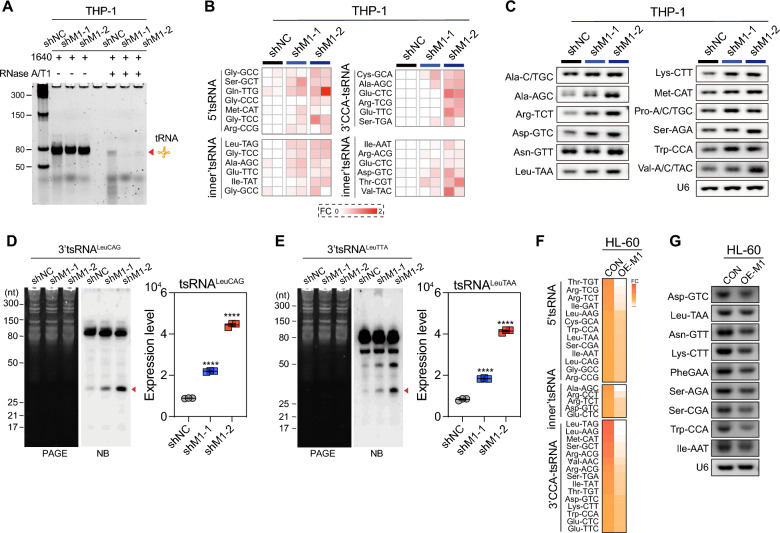
METTL1 knockdown promotes tsRNA biogenesis. **A** tRNA degradation levels on PAGE gels under RNase A/T1 in THP-1 cells upon METTL1 knockdown. **B** High-throughput small RNA sequencing analysis of tsRNA expression in THP-1 cells upon METTL1 knockdown. **C** Representative bands of tsRNA levels examined by RT-PCR in THP-1 cells upon METTL1 knockdown. **D**, **E** Northern blot analysis of 3′tsRNA^LeuCAG^ and 3′tsRNA^LeuTAA^ in THP-1 cells upon METTL1 knockdown. Left: NB images. Right: quantification data. **F** High-throughput small RNA sequencing analysis of tsRNAs expression in THP-1 cells upon METTL1 overexpression. **G** Representative bands of tsRNAs levels examined by RT-PCR in THP-1 cells upon METTL1 overexpression. shNC: METTL1 control, shM1: METTL1 knockdown, CON: METTL1 control, OE-M1: METTL1 overexpression. Data were presented as mean ± SEM (Student’s t test, ****p < 0.0001)

To investigate how the knockdown of the METTL1 gene affects the tRNA spectrum, total tRNAs isolated from METTL1 knockdown AML cells and METTL1 control AML cells were treated with RNase A/T1 (a mixture of Rnase A and Rnase T1, in which Rnase A specifically cleave RNA at C and U residues and Rnase T1 specifically cleaves RNA at G residues). The results showed that the knockdown of METTL1 increased the susceptibility of tRNA to degradation by Rnase A/T1 (Fig. 7A), thereby accounting for the altered tRNAs observed in AML cells caused by METTL1 knockdown. As a new type of regulatory small non-coding RNAs, tRNA-derived small RNAs—tsRNAs (also known as tDRs or tRNA derived fragments, tRFs) showed important roles in various fundamental biological conditions and in regulating tumorigenesis in multiple cancers, such as breast cancer, hematological malignancies, and lung cancer [46–48]. The biogenesis of tsRNAs is sensitive to various cellular stresses and determined by the secondary structure and the methylome of their precursor tRNAs, which can expose the cleavage site to multiple Rnases to promote the biogenesis of tsRNAs [49]. In this study, we found that the knockdown of METTL1 decreased the m^7^G levels in total tRNAs and promoted the degradation of tRNA under the digestion of Rnase A/T1, which might facilitate the biogenesis of tsRNA in AML cells. To explore whether the knockdown of METTL1 could increase the overall levels of tsRNA in AML cells, we performed the high-throughput small noncoding RNA sequencing experiment between METTL1 knockdown and METTL1 control AML cells, the results showed that the levels of some tsRNAs, including 5’tsRNA, inner’ tsRNAs, and 3’CCA-tsRNAs are remarkably increased in METTL1 knockdown AML cells (Fig. 7B), which were further confirmed by northern blot and RT-PCR (Fig. 7C–E). Conversely, high-throughput small noncoding RNA sequencing and RT-PCR experiments demonstrated a corresponding reduction in the majority of tsRNAs in AML cells with METTL1 overexpression (Fig. 7F, G). However, when transfected with the isolated tsRNAs (30–40 nt) fragments from METTL1 knockdown cells into AML cells, the overall translation efficiency in AML cells was not affected (Additional file 4: Fig. S4), indicating that the molecular regulation mechanism of tsRNAs might not involve in cellular nascent protein synthesis as tRNAs in AML cells and more investigations should be performed to unveil the underline mechanism of tsRNA in AML leukaemogenesis. Taken together, the above results demonstrate that METTL1-mediated m^7^G modification on tRNAs plays an important role in the regulation of tRNA stability and tsRNA biogenesis in AML cells, which might further affect the cell viability and leukaemogenesis of AML.

## Discussion

Transfer RNAs (tRNAs) are the crucial adapter molecules during protein synthesis for deciphering mRNA codons and translating the corresponding amino acids [50]. By far, numerous post-transcriptional modifications have been discovered in tRNAs [51], which are important for tRNA higher structure formation, tRNA stability, and decoding function [52–54]. Moreover, the maladjusted tRNA modifications can lead to a wide range of pathological consequences such as mitochondrial diseases, neurological disorders, and cancers [55]. As one of the most prevalent tRNA modifications, m^7^G is mainly located at nucleotide position 46 in the variable loop of a large subset of tRNAs, and is catalyzed by the METTL1/WDR4 complex in humans [56]. In recent years, an increasing number of studies revealed that dysregulations of METTL1/WDR4-mediated tRNA m^7^G modification played an important biological function in human malignancies such as liver cancer, colorectal cancer, and lung cancer, including tumor progression and chemotherapeutic sensitivity which served as a promising candidate target for the prevention and treatment of tumors [27, 33, 35, 57, 58]. Recently, Orellana et al revealed that the METTL1/WDR4 complex could regulate the oncogenesis of many types of human cancers, such as breast cancers, glioblastomas, certain sarcomas, and AML via tRNA Arg^TCT−4–1^ mediated translation bias of cell growth associated mRNAs, and emphasis on the ubiquitous role of METTL1 across various tumors [35]. However, the specific functions and molecular mechanisms of METTL1 in AML are not their priority to illustrate.

In this study, we conducted a comprehensive investigation into the expression level of METTL1 and WDR4 and its correlation with prognosis in AML patients. We also revealed that the METTL1/WDR4 complex promoted cell proliferation, inhibited cell apoptosis in AML cells, suggesting that METTL1 acts as a tumor accelerator in AML. Additionally, we also found that METTL1 knockdown increased drug susceptibility to cytarabine in AML cells. Recent studies have demonstrated the synergistic effects of combining epigenetic modifications with targeted therapies for AML treatment [4, 10], supporting the idea that targeted epigenetic modifications, such m7G, combined with traditional anti-tumor medicine might be a potential synergistic treatment strategy for AML therapy.

Mechanistically, by performing O-propargyl-puromycin (OPP) labeling assay, a reduction level of nascent protein synthesis has been found in METTL1 knockdown AML cells, suggesting the decreased global mRNA translation caused by METTL1 knockdown. Orellana EA et al. revealed that tRNA^Arg−TCT^, one of the m^7^G-modified tRNAs, plays a crucial role in METTLI-mediated mRNA translation variation [35]. In our study, we found a decreased translation efficiency in AML cells which were transfected with total tRNAs extracted from METTL1 knockdown cells, suggesting that changes in global mRNA translation under METTL1 knockdown may be caused by variations in the tRNA spectrum. Whether tRNA^Arg−TCT^ plays a major role in inhibiting cellular translation efficiency in AML cells is still under investigation. Moreover, the present study confirms that METTL1 is involved in the maintenance of tRNA stability in AML cells and that METTL1 knockdown results in a reduction of various tRNA abundance and elevated the levels of tsRNAs. The emerging evidence shows that abnormal expression of individual tsRNAs is involved in cancer biology [25, 46, 47, 49, 59, 60]. However, we did not observe significant alteration of cellular translation efficiency when transfected in vivo isolated tsRNAs from METTL1 knockdown cells into AML cells, suggesting that the molecular regulatory mechanisms of tsRNAs in AML might beyond regulating cellular nascent protein synthesis. In addition to tRNA, we also observed significant decrease of m^7^G abundance on mRNAs, however, whether METTL1-mediated mRNA m^7^G modification metabolism also participated in leukaemogenesis of AML is still subject to further experimental confirmation.

In our study, we found a negative correlation between DNA methylation levels and mRNA expression of METTL1 in AML patients and downregulation of METTL1 expression upon *Tet* inhibitor (Bobcat339 hydrochloride) [61] treatment, however, the exact molecular mechanism of DNA methylation modifiers in regulating METTL1 expression still need further investigation. Moreover, the present study shows that METTL1 modulates the expression of WDR4 but not vice versa. The collaborative expression between METTL1 and WDR4 was identified among various cancers [35]. Recent structural studies confirmed that METTL1 and WDR4 cooperate to recognize RNA substrates and catalyze methylation, in which WDR4 serves as a scaffold for METTL1 and tRNA T-arm [31, 32]. However, how METTL1 regulates the WDR4 expression at protein levels is still unclear. Interestingly, we found that the level of Gm, m^5^C, I, and Ψ modification on tRNA or mRNA were altered with m^7^G abundance declines in AML cells after METTL1 knockdown. We previously reported that the abundances of different types of RNA modifications exist in extensive correlation in human sperm and mammalian tissue RNAs, such as m^5^C vs m^2^G and m^5^C vs m^7^G [38, 62]. Although the underlying mechanisms have not been completely elucidated, the observation of non-METTL1-mediated RNA modifications abundance alteration in this study provides new research insights into different RNA modification cooperation in regulating leukaemogenesis of AML.

## Conclusions

In summary, our study demonstrates that the up-regulation of METTL1/WDR4 in AML patients increases the abundance of tRNA m^7^G modification, which promotes the leukaemogenesis of AML cells by facilitating the overall cellular translation efficiency. providing a potential therapeutic target for AML.

### Supplementary Information

**Additional file 1: Figure S1.** METTL1/WDR4 were upregulated in AML patients. (A-B) Comparison of METTL1 and WDR4 expression in healthy donors and WHO subtypes of AML patients from our center. (C-D) Comparison of METTL1 and WDR4 expression in healthy individuals (NC) and WHO subtypes of AML patients from GEO dataset. Data were presented as mean ± SD (Student’s t test, *p < 0.05, **p < 0.01, ns: not significant).

**Additional file 2: Figure S2.** METTL1 knockdown inhibits the survival of human AML cells. (A-B) Representative images of cell apoptosis in METTL1 knockdown (shM1) and METTL1 control (shNC) AML cells while treated with cytarabine (CYT) for 48 h. A: MOLM-13 cells. (B): THP-1 cells.

**Additional file 3: Figure S3.** The abundance of cellular RNA modifications on tRNA and mRNA in METTL1 knockdown and METTL1 control THP-1 cells. (A) Comparison of modification level on tRNA in METTL1 knockdown (shM1) and METTL1 control (shNC) THP-1 cells. From left to right, they are A, U and C modifications, respectively. (B) Comparison of modification level on mRNA in METTL1 knockdown (shM1) and METTL1 control (shNC) THP-1 cells. From left to right, they are A, U and C modifications, respectively. Data were presented as mean ± SD (Student’s t test, *p < 0.05, **p < 0.01, ***p < 0.001).

**Additional file 4: Figure S4.** METTL1 overexpression leads to decreased tsRNA biogenesis. (A) The level of OPP means fluorescence intensity (MFI) in THP-1 cells transfected with tsRNAs extracted from METTL1 knockdown (shM1) and METTL1 control (shNC) THP-1 cells, respectively.

**Additional file 5: Table S1.** Sequence of primer and DIG labelled probes.

## Data Availability

The datasets used and/or analyzed during the current study are available from the corresponding author on reasonable request.

## Abbreviations

m^7^G: N7-methylguanosine
m^6^A: N6-methyladenosine
Ψ: Pseudouridine
METTL1: Methyltransferase complex-methyltransferase like 1
WDR4: WD repeat domain 4
tRNAs: Transfer RNAs
tsRNAs: TRNA derived small RNAs
LC–MS: Liquid chromatography-coupled mass spectrometry
ATCC: American Type Culture Collection
GEO: Gene Expression Omnibus
TCGA: The Cancer Genome Atlas
GO: Gene Ontology
PBMCs: Peripheral blood mononuclear cells
BMMCs: Bone marrow mononuclear cells
shMETTL1: METTL1 knockdown
shNC: METTL1 negative control
OE-METTL1: METTL1 overexpression
OE-WDR4: WDR4 overexpression
MRM: Multiple reaction monitoring
DEP: Differentially expressed proteins
CYT: Cytarabine
OPP: O-propargyl-puromycin
MFI: Mean fluorescence intensity
