# Supplementary figures and images for: METTL1 mediated tRNA m7G modification promotes leukaemogenesis of AML via tRNA regulated translational control
Source: Exp Hematol Oncol. 2024 Jan 24;13:8. doi: 10.1186/s40164-024-00477-8 (PMC10807064; doi:10.1186/s40164-024-00477-8)

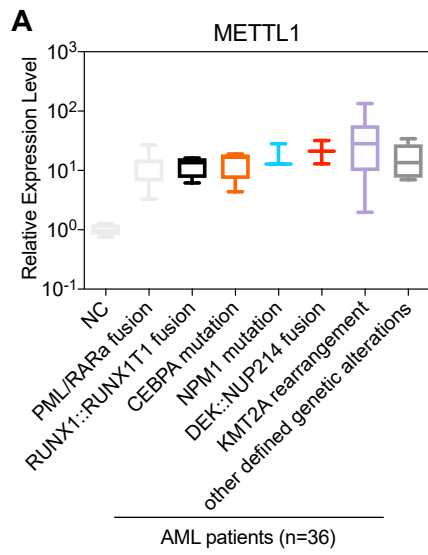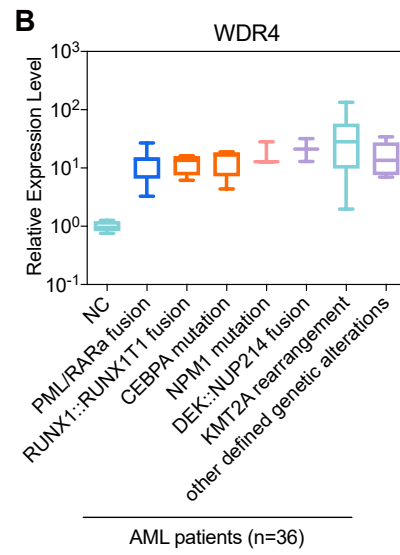

Supplement: Supplementary file 1 — Additional file 1: Figure S1. METTL1/WDR4 were upregulated in AML patients. (A-B) Comparison of METTL1 and WDR4 expression in healthy donors and WHO subtypes of AML patients from our center. (C-D) Comparison of METTL1 and WDR4 expression in healthy individuals (NC) and WHO subtypes of AML patients from GEO dataset. Data were presented as mean ± SD (Student’s t test, *p < 0.05, **p < 0.01, ns: not significant). [file 40164_2024_477_MOESM1_ESM.pdf]

**A**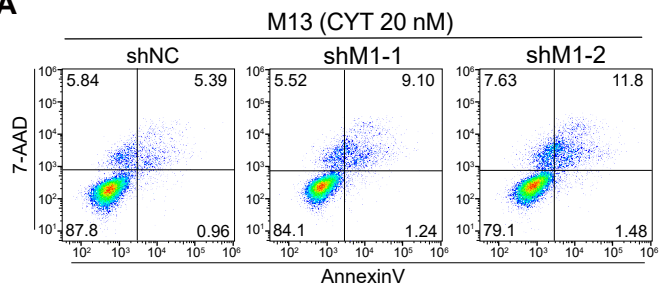**B**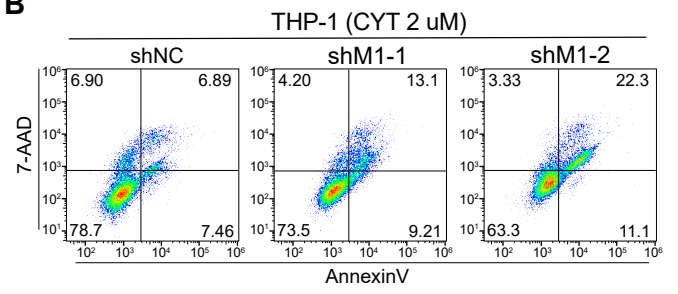

Supplement: Supplementary file 2 — Additional file 2: Figure S2. METTL1 knockdown inhibits the survival of human AML cells. (A-B) Representative images of cell apoptosis in METTL1 knockdown (shM1) and METTL1 control (shNC) AML cells while treated with cytarabine (CYT) for 48 h. A: MOLM-13 cells. (B): THP-1 cells. [file 40164_2024_477_MOESM2_ESM.pdf]

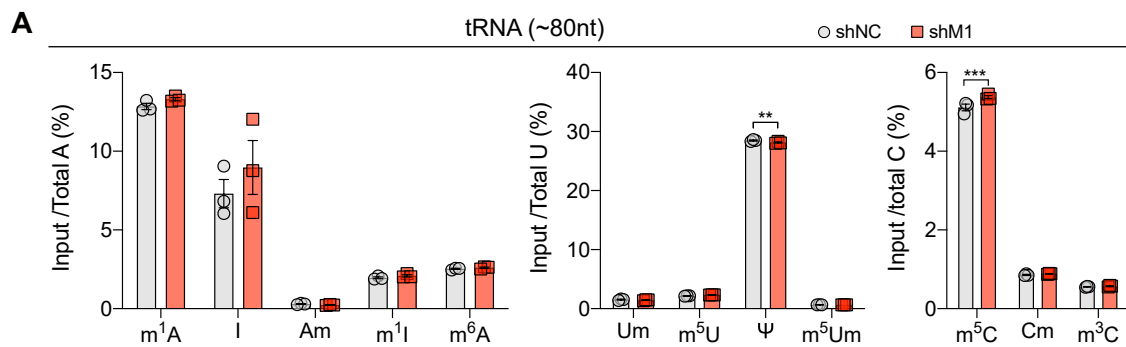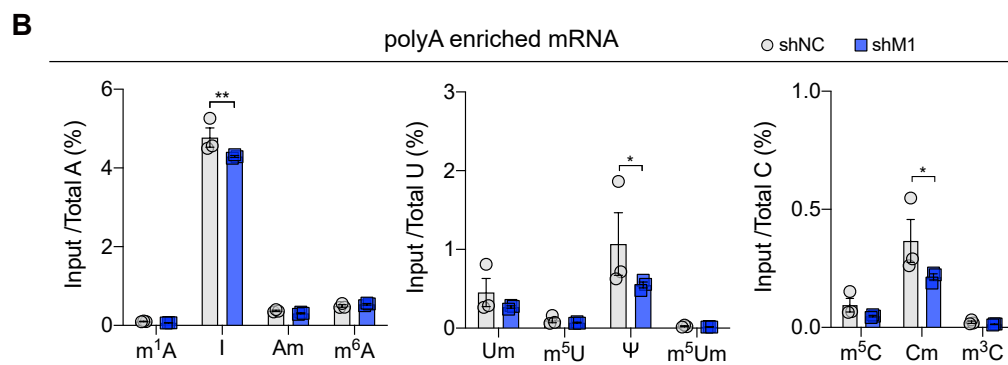

Supplement: Supplementary file 3 — Additional file 3: Figure S3. The abundance of cellular RNA modifications on tRNA and mRNA in METTL1 knockdown and METTL1 control THP-1 cells. (A) Comparison of modification level on tRNA in METTL1 knockdown (shM1) and METTL1 control (shNC) THP-1 cells. From left to right, they are A, U and C modifications, respectively. (B) Comparison of modification level on mRNA in METTL1 knockdown (shM1) and METTL1 control (shNC) THP-1 cells. From left to right, they are A, U and C modifications, respectively. Data were presented as mean ± SD (Student’s t test, *p < 0.05, **p < 0.01, ***p < 0.001). [file 40164_2024_477_MOESM3_ESM.pdf]

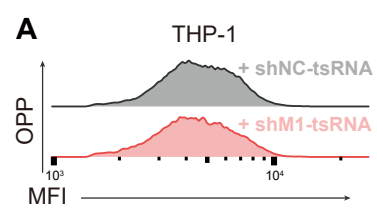

Supplement: Supplementary file 4 — Additional file 4: Figure S4. METTL1 overexpression leads to decreased tsRNA biogenesis. (A) The level of OPP means fluorescence intensity (MFI) in THP-1 cells transfected with tsRNAs extracted from METTL1 knockdown (shM1) and METTL1 control (shNC) THP-1 cells, respectively. [file 40164_2024_477_MOESM4_ESM.pdf]
